# Extremophiles and Extremophilic Behaviour—New Insights and Perspectives

**DOI:** 10.3390/life14111425

**Published:** 2024-11-05

**Authors:** George N. Angelakis, Chrysianna Psarologaki, Stergios Pirintsos, Kiriakos Kotzabasis

**Affiliations:** 1 Department of Biology, University of Crete, Voutes University Campus, GR 70013 Heraklion, Crete, Greece; 2 Faculty of Geosciences, Utrecht University, 3508 TC Utrecht, The Netherlands; 3 Faculty of Biology and Psychology, Georg-August University of Göttingen, Wilhelm-Weber-Straße 2, 37073 Göttingen, Germany; 4 Botanical Garden, University of Crete, Gallos University Campus, GR 74100 Rethymnon, Crete, Greece

**Keywords:** extremophiles, niche, astrobiology, astrobiotechnology, origin of life, panspermia, terraforming

## Abstract

Extremophiles, throughout evolutionary time, have evolved a plethora of unique strategies to overcome hardships associated with the environments they are found in. Modifying their genome, showing a bias towards certain amino acids, redesigning their proteins, and enhancing their membranes and other organelles with specialised chemical compounds are only some of those strategies. Scientists can utilise such attributes of theirs for a plethora of biotechnological and astrobiological applications. Moreover, the rigorous study of such microorganisms regarding their evolution and ecological niche can offer deep insight into science’s most paramount inquiries such as how life originated on Earth and whether we are alone in the universe. The intensification of studies involving extremophiles in the future can prove to be highly beneficial for humanity, even potentially ameliorating modern problems such as those related to climate change while also expanding our knowledge about the complex biochemical reactions that ultimately resulted in life as we know it today.

## 1. Extremophiles and Extremophilic Behaviour

The term “extremophile” is used to describe organisms that have managed to not only survive but thrive at the most extreme ranges of environmental variables on Earth. MacElroy was the first scientist to ever use this term in 1974 [1]. However, one should always keep in mind that terms like “extreme” are somewhat human-centred. To avoid inaccuracies, it is better to describe extreme conditions of life, such as high or low temperatures, pressure, acidity, or dryness, which would be harmful to other organisms except for extremophiles [2]. In addition, “extremophilic behaviour” is expanded beyond extremophiles, a trait that various organisms of different taxonomic groups can display when faced with extreme conditions.

Extremophiles offer a unique way to explore questions about the origins and limits of life. They help scientists search for habitable planets and potential alien life, and they provide valuable insights for developing useful biotechnological applications.

## 2. Survival Strategies Under Extreme Conditions

An abundance of data already exists to support that extremophiles’ adaptation strategies in extreme environments mainly concern the augmentation or stabilisation of each group of extremophiles’ enzymatic activity and membrane consistency.

### 2.1. Survival Under Extreme High Temperatures

Undoubtedly, thermotolerant and/or thermophilic microorganisms, able to sustain themselves and reproduce in temperatures higher than 60 °C, need to employ a variety of strategies that mitigate or bypass common problems that high temperatures cause, such as the annealing of nucleic acids, the denaturation of proteins, or the lack of appropriate stability of cell structures.

In order to achieve that, it has been shown that thermophiles tend to have much more compact and smaller genomes than those of their non-thermophilic counterparts [3]. Moreover, they show a strong bias towards certain amino acids or certain amino acid patterns [4] which can be used to synthesise derivatives beneficial to their survival [5]. Furthermore, studies concerning the structure of histones reveal a plethora of histone paralogs that co-exist in thermophilic archaea that increase DNA affinity and tetramer stability [6], thus increasing DNA’s tolerance to temperature-driven annealing.

When it comes to maintaining enzyme stability, thermophiles have employed various strategies that partially depend on the final destination of enzymes (intra- or extracellular). The stability of enzymes is reliant on factors such as the presence of compatible solutes [7,8,9,10]; their interactions with other cellular components [2]; possible amino acid substitutions [11], with a final intention of increasing the number of hydrogen bonds and salt bridges [12]; or, lastly, interactions with other proteins [13]. Recently, scientists have also proposed that heat shock proteins had a paramount role in the creation of early cellular membranes [14].

Lastly, the membrane lipids of some bacteria contain a unique glycerol ether lipid called 15,16-dimethyl-30-glyceryloxy-triacontanedioic acid. Unlike the ester lipids found in mesophiles, this glycerol ether lipid is thought to greatly enhance membrane stability against hydrolysis at high temperatures, thus protecting cell components from heat-induced damage in extreme thermal environments [15].

### 2.2. Survival Under Extreme Low Temperatures

Psychrophiles are divided into two, somewhat overlapping, categories: psychrophiles sensu stricto that exhibit optimal growth below 15 °C and psychrotolerant organisms that can survive in sub-zero temperatures yet exhibit optimal growth around 20 °C [16,17]. It is argued that psychrophiles represent the most abundant group of extremophiles on Earth [16,18].

The biggest challenge psychrophiles faced in adapting to their environment was maintaining a proper metabolic rhythm and counteracting the slowing effects of low temperatures on their biochemical reactions. In order to achieve that, the enzymes of such microorganisms exhibit low-activation enthalpy and highly specific activity at low temperatures [19], achieving an optimal balance between activation energy and enzyme specificity.

Another challenge for these microorganisms is the high viscosity of their environment. To adapt, psychrophiles increase the content of unsaturated and methyl-branched fatty acids [20,21], produce lipids with shorter acyl chains, and increase the content of large lipid head groups, proteins, and non-polar carotenoid pigments [22]. However, the validity of these findings is still debated due to a lack of extensive research across multiple species [18]. Transcription and translation are also temperature-sensitive steps and, hence, psychrophiles have modified them accordingly. At colder temperatures, DNA strands form stronger interactions in the double helix and supercoiled state, hindering unwinding and RNA polymerase access [23]. Additionally, low temperatures promote the formation of RNA secondary structures that can interfere with translation. To overcome these challenges, psychrophiles likely rely on nucleic acid-binding proteins, including RNA helicases, to alleviate the impact of low temperatures on nucleic acid structure and function [24,25].

Lastly, a comparative study between the cold-tolerant and non-tolerant isolates of the same genus of bacteria has revealed major differentiations in G/C content, an amino acid bias, and differentiations in the representation of specific cellular functions [26].

### 2.3. Adaptations to Extreme High Acidity

Regarding acidophiles, there are still large gaps in their adaptation mechanisms. In a recent study, scientists were able to propose a list of candidate genes and associated proteins that may be able to assist microorganisms in overcoming difficulties that arise in acidic environments [27].

Additionally, solid evidence supports the notion that the genomes of acidophiles, in the same manner as psychrophilic microorganisms, exhibit tremendous genome flexibility. A diverse number of integrative conjugative and transposable elements of various sizes have been found to populate the genomes of acidophilic bacteria and archaea and are hypothesised to aid their adaptability in such extreme environments [28].

### 2.4. Adaptations to Extreme High Alkalinity

Alkaliphiles consist of one of the most understudied groups of extremophiles. Nonetheless, individual studies on certain alkaliphiles have already provided us with exciting data on their unique adaptations. For instance, alkaliphiles, such as *Bacillus clausii* KSM-K16 and *Bacillus alcalophilus* AV1934, exhibit nonconventional cation-coupled flagellar motors, with the parallel utilisation of both Na^+^ and K^+^ by the MotP subunit [29,30].

Due to the environmental conditions in which alkaliphiles reside, maintaining proper cellular respiration can prove to be a difficult task. For that reason, species like *Pseudomonas alcaliphila* AL15-21T [31] and *Bacillus pseudofirmus* OF4 [32] have increased the production of electron-retaining soluble substances such as cytochrome c [31] or cell wall surface proteins like SlpA [32] that play the role of electron and proton condensers [31].

Moreover, microorganisms such as *Shewanella oneidensis* MR-1 [33] and various *Tissierella*, *Clostridium*, and *Alkaliphilus* spp. have been shown to employ the use of flavins, most likely a riboflavin analogue, to reduce metals in the environment by using iron as a final electron acceptor, playing a key role in extracellular metal reduction [34,35].

In addition, alkaliphiles have demonstrated a strong inclination for protein structures or other soluble substances that are able to capture protons and thus prevent proton loss from the cell. It is argued that through this strategy of the localisation of proteins and substances in or around membranes, alkaliphiles are able to retain the desired intracellular pH level [36,37,38].

### 2.5. Adaptations to Extreme High Pressures

Piezophiles comprise a special case of extremophiles in the sense that they do not usually exist as a separate group but as an extension of other extremophilic subgroups [39]. In the broader sense though, piezophiles are characterised as microorganisms that are able to thrive under pressure equal to or above 10 MPa [40].

These microorganisms need to possess adapted proteins and enzymes able to function under such augmented compression rates [41]. Moreover, high pressure is one of the main culprits for the presence of relatively high concentrations of reactive oxygen species (ROS) via the creation and/or sustaining of an oxidation/reduction imbalance [42,43].

Recent studies on the piezophile *Moritella profunda* [44] and the hyperpiezophile *Moritella yayanosii* [44] have confirmed the long-standing hypothesis that proteins from piezophiles sustain high compressibility by possessing cavities of a larger volume compared to their homologous non-piezophilic derived proteins [44,45]. These cavities help them maintain lower pressure stability [46] because the system volume is lowered when water enters these cavities. What remains puzzling still is that such cavities have also been associated with lower unfolding pressures [44,45].

The nature of the changes on the protein level is multifaceted. It has been adequately demonstrated that the presence of hydrophilic and polar amino acid groups drives piezophilic protein adaptation in thermo-piezophilic microorganisms, exhibiting a higher preference for hydrophobic and non-polar amino acids [47]. Moreover, other species, mainly psychro-piezophiles, have been shown to employ various intrinsic pre- and post-transcriptional molecular adaptations to counteract such high pressures [48,49]. In any case, further investigations are needed on such adaptive mechanisms.

### 2.6. Adaptations to Ionising and UV Radiation

A group of extremophiles that is, more than often, neglected are those that are able to sustain extremely high amounts of radiation, whether it is ionising or UV. Especially, when trying to draw parallels between extremophiles and possible candidates in the theory of panspermia, one should always keep in mind the extremely high levels of radiation those microbes would have to be exposed to for large periods of time [50]. Nonetheless, most studies that aim to pinpoint the exact mechanisms that confer such resistance are still scarce [51,52,53,54].

A long-standing theory suggests that resistance to radiation may likely be found in numbers rather than in individual cellular mechanisms. Experiments, even in non-extremophilic species such as *Staphylococcus aureus*, suggest that bacterial tolerance to photodynamic treatment (PDT) is possible if a large bacterial suspension (instead of singular colonies) is subjected to multiple cycles of sublethal exposures and subsequent re-culture [55]. It is almost a given that no matter the actual strategies that drive such a resistance, an important component of that is the building of a gradual resistance to radiation after prolonged exposure to sublethal amounts of it [56,57].

### 2.7. Adaptations Related to Industrial Settings

In addition to their unique adaptations, extremophiles exhibit traits such as xerophilicity and metallophilicity that are highly advantageous in industrial settings. For example, newly identified strains of *Acidithiobacillus ferrooxidans*—which are simultaneously psychrotolerant, acidophilic, and metallophilic—show great promise for the metal bioleaching industry [58]. Furthermore, there is growing interest in modifying extremophiles to produce a range of compounds, from nutritional supplements and metabolic chemicals [59,60] to fuels [61]. As a result, extremophiles with multiple specialised properties are becoming increasingly valuable as the range of extreme industrial applications expands [62]. The enzyme industry, too, is leveraging both newly discovered and well-known extremophiles to mass-produce proteins like glycosidases, esterases, and proteases [63], demonstrating that these organisms can prosper in industrial environments and offer various valuable biotechnological products.

## 3. Extremophiles in Astrobiological Applications

### 3.1. Applications in Space Missions

Since the deployment of the International Space Station (ISS), a plethora of astrobiology experiments have been conducted. Recent studies in the field of space microbiology are concerned with various subjects including, but not limited to, creating effective microbial monitoring systems aiming at both the ISS environment as well as the microbiome of the deployed astronauts [64,65,66,67], expanding upon the resilience of microorganisms in space conditions [68,69,70], or even characterising novel species [71].

It is often forgotten that the ISS constitutes an extreme environment for microorganisms as well. The station is a hermetically closed, micrοgravitational, high radiation, high CO_2_ system whose available air flow is also subjected to HEPA filters [65,66]. It is true, nonetheless, that microorganisms not classically categorised as extremophiles have been found inside the station, potentially questioning the robustness of the current terminology of extremophiles.

While assessing such data, it is important to keep in mind that a subset of those microorganisms may have derived from clean rooms where assemblies of ISS components have taken place, and thus “environmental pressures” have already been applied to them prior to the launch of the part. Similar cases involving such isolations have already been well established and analysed [72,73].

*Deinococcus radioendurans* constitutes an exemplary model organism that is widely used to study the endurance of microorganisms in space. In a 1-year-long study, scientists aimed to pinpoint the exact molecular repertoire differentiations that exposure to space conditions causes [58]. *D. radioendurans* cells seem to employ a plethora of strategies to alleviate cell stress. A classic example of this coordinated response is the expression of the UvrABC endonuclease excision repair mechanism that “undoes” nucleotide mutations caused by exposure to UV radiation.

### 3.2. Extremophiles: Unravelling Habitability Beyond Earth

Up until recently, in an effort to apply ecological models to the field of astrobiology, various habitability models co-existed [74,75,76], making it difficult for studies to expand upon each other or even reach any form of consensus. In 2021, a consortium of various scientists around the world aimed to tackle this problem while also expanding upon the existing Habitat Suitability Models (HSMs), paving future directions in the field of exoplanetary habitation [77].

A theory that has been put forward and gained increasingly more ground suggests that planet habitability is not a binary question [77]. As the authors put it, “Does a planet that hosts a one-micron cube that is habitable to a single microorganism classify as a habitable planet?”. According to them, the answer is yes. Even if this assumption is not ubiquitously accepted, the fact remains that microorganisms have consistently proven their higher levels of resilience and adaptability in “niche” environments in contrast with other living organisms such as plants and animals.

In an effort to study the habitability of other planets, scientists actively seek or simulate extreme environments on Earth that approximate those of other planets. The studying of various niche ecosystems in terms of their level of habitability is considered a paramount source of information. Various studies have been carried out in places like the Jezero and Gusev Craters [77,78] and the Jökulhlaup Deposits of Southern Iceland [79]. Their results suggest that whether an environment is habitable or not almost solely depends on the presence of liquid water and the presence of chemical elements such as C, S, and Fe that could also be used as an energy source [80] in a manner parallel to what chemolithotrophic organisms have already demonstrated [81,82]. Consequently, potential analogous Martian sites that contained a similar set of conditions may have harboured microbial life in the past [80].

Lastly, simulated environments that closely resemble those observed in potentially habitable exoplanets have been created in order to study the limits of the growth and endurance of various microorganisms. MARSBox (Microbes in Atmosphere for Radiation, Survival, and Biological Outcomes Experiment) was a microorganism-containing platform that was launched in September 2019 in the middle stratosphere of Earth in an effort to simulate radiation levels observed in the equatorial surface of Mars. The results that were obtained strengthened the ongoing hypothesis that pigmented fungi may prove to be resistant to the Martian surface [83].

### 3.3. Harnessing Extremophiles: Strategies for Planetary Terraforming

In the wider research field of astrobiology, terraforming is considered the next logical step following research concerning the potential habitability of other planets. Terraforming is defined as the hypothetical process of deliberately modifying or de novo creating the environmental conditions of a planetary body to closely resemble the environment observed on Earth and thus make it habitable for humans.

Even though scientists have suggested that hypobaric plant species such as *Arabidopsis thaliana* can be used as secondary terraforming modules in CO_2_-rich atmospheres such as the one observed on Mars [84], the general consensus is that extremophilic microorganisms should be the primary means of terraforming.

For over twenty years, extremophilic cyanobacteria have been suggested as possible tools for terraforming because they can survive extreme conditions like dryness, cold, heat, and high salt levels, and they can also photosynthesize [85]. Most importantly, scientists have proven that certain cyanobacteria, namely ones of the *Nostoc* spp., are able not only to withstand but grow during freeze–thaw cycles in the range of −12 °C to 26 °C when found in the form of biological soil crusts (BSCs). Moreover, it should be noted that BSCs can be further enhanced by aerobic anoxygenic phototrophic bacteria (AAPBs) such as *Alphaproteobacteria* and *Gammaproteobacteria* [86].

Another proposal that has gained a lot of support in the last few decades explores the possibility of using algae to terraform exoplanets due to their ability to survive in Mars-like conditions for prolonged periods of time [87,88]. The micro-ecosystem of lichens that is created by the symbiosis of mycobiont and photobiont parties has been proven to be viable even under prolonged desiccation and extremely low (−196 °C) [89] and high (+70 °C) temperatures [89], in extreme high UVB radiation [89], in vacuum (10^−7^ to 10^−4^ Pa) [90], and under cosmic ionising radiation (up to 190 mGy) [91] and solar extraterrestrial electromagnetic radiation (up to 6.34 × 10^8^ J m^−2^) [92].

### 3.4. Extremophiles: Unveiling Insights into Life’s Origins

Many scientific theories have been proposed over the years regarding the origin of life. Extremophiles devise a central piece to some of those theories owing to the fact that by definition, they extend the widest possible spectrum of conditions that are able to sustain life. The most famous of those theories is the theory of panspermia and its derivative theories.

#### 3.4.1. Extremophiles and the Theory of Panspermia

As one may infer, sometimes extreme conditions on Earth have the potential to approach the ones observed in deep space. Some key differences do exist though, since space conditions can prove to be even harsher and more variable. If one wishes to consider extremophiles as the precursors to the proof of the theory of panspermia, one needs to consider the rapid switches in extremes that can be observed in space.

Radiopanspermia suggests a mechanism for interstellar transfer of life, where organisms may be transported on dust particles propelled by radiation pressure from stars, which acts as the dominant mechanism for accelerating organism-laden material of grain size out of the first planetary system and decelerating it into the second system. However, this form of transport poses significant challenges as ultraviolet light is identified as the dominant mechanism for inactivating organisms in space [93].

Stellar bodies like comets are regarded as paramount candidates in the spreading of life according to the theory of lithopanspermia due to their protective nature, which can shield microbial life from harmful ultraviolet photons and cosmic ray particles during interstellar travel [94,95]. Moreover, the possibility that potential microorganisms could reside inside comets, increasing their potential to endure the rigours of interstellar travel, is supported by the evidence of microorganisms thriving within rocks and forming subterranean biospheres independent of light or external nutrients [96,97]. Comets harbour distinct environments in their comae that can vary widely in properties [3,98]. A comet’s variability is further exacerbated by phenomena such as the uneven distribution of necessary compounds like water and the high variability in outgassing fluxes, heavily impacting the comet’s topography and local conditions, thus causing major differentiations even a few hundred metres apart [99].

Given the complexity of these environments and our limited understanding, pinpointing the exact conditions for life on a comet might seem pointless. However, identifying the possible range of conditions that could support life helps us explore whether extremophiles and other microorganisms could survive there. This approach supports the panspermia theory, which suggests that if life exists across the universe, it must adapt to specific environmental limits.

#### 3.4.2. Could Extremophiles Be Irrelevant to the Origin of Life?

Cleaves and Chalmers (2004) made the bold hypothesis that extremophiles may be irrelevant to the origin of life. This article was published during a heated debate on the validity of panspermia, lithopanspermia, and the importance of extremophiles in these theories. The authors argued against the relevance of extremophiles for two main reasons. First, they claimed that the harsh conditions of interplanetary travel and arrival, such as high impact forces, UV radiation, intense heat, and the shock of landing [100,101], would require complex adaptations that early life forms likely lacked. Second, they argued that there is not enough evidence to support the idea that all the extreme environments where life exists today could have originally fostered the emergence of life [102].

Nonetheless, since then, studies have been published that expand our knowledge on these topics and hint at quite the opposite. Regarding their first argument, new data have arisen that provide a set of equations that mathematically allow the interplanetary transportation of microorganisms as a viable possibility [103]. Moreover, in recent years, simulations of the conditions of interplanetary travel [104,105] and the subsequent collision into Earth-like or early Earth-like environments [106,107,108] do extend the possible set of conditions that would allow such a case. When it comes to their second point, the abundance of studies before and since then goes to show that extreme environments on Earth are indeed our best tool to study the origin of life hypotheses [109,110,111]. It has been proven time and time again that those environments are our closest in situ approximation of early Earth conditions and the organisms that reside or resided there (by definition, all of them extremophiles) are our best tool to understand the limits of life [112,113]. Regardless of how life emerged, it, nonetheless, had to sustain itself in environments like those [114,115].

New data increasingly links extremophiles with theories on the origin of life. Studying extremophiles is our most reliable way to move beyond speculation about how life began. It is possible that extremophiles were the first organisms to emerge on any planet capable of supporting life, regardless of how or how often they appeared, or whether they travelled between planets. Extremophiles are not a fixed group of microorganisms; they are a diverse and growing category defined by human-centred limits, making their role in these theories difficult to dismiss.

#### 3.4.3. Did Extremophilic Life Emerge near Hydrothermal Vents?

In the last few decades, submarine hydrothermal vents have been proposed as a prime topological site able to support the emergence of the first forms of life. This theory has come to be presented as an extension or alteration of the primordial soup theory by proposing that an H_2_-dependent chemistry of transition metal sulphide catalysts in a hydrothermal vent setting could be enough to sustain the very first forms of life [116].

These vents are classified either as “black smoker” or “lost city” vents, and their main difference is how they reach their highest temperatures and what those maximum temperatures are. Black smoker vents are heated by magma beneath the ocean floor, while lost city vents are powered by a chemical process called serpentinization.

Serpentinization is a complex reaction that transforms minerals like olivine, pyroxene, and amphiboles into serpentine minerals. For example, olivine, an unstable mineral, undergoes this reaction when it interacts with water, producing serpentine, magnetite, and hydrogen [117]. This process also occurs with other similar minerals and is considered a simple and effective way to create energy through proton and redox gradients. Unlike other theories of how life began, this reaction did not require early biochemical systems to create specific reactions but simply used the existing ones [118,119]. If life emerged using this energy, it likely happened in freshwater hydrothermal fields rather than submarine vents [120].

One should always bear in mind that this theory, regardless of the variation in the proposed pathways, takes as a prerequisite that the first ever life forms to emerge would have to tolerate high temperatures and pressure, thus de facto characterising those very first microorganisms as extremophiles. Further research into if and how modern extremophiles adapt to such environments will provide paramount insight into how ancient extremophiles could have done the same.

## 4. Extremophiles as De Facto Organisms or as Response of Common Organisms to Extreme Conditions

**Figure 1:**
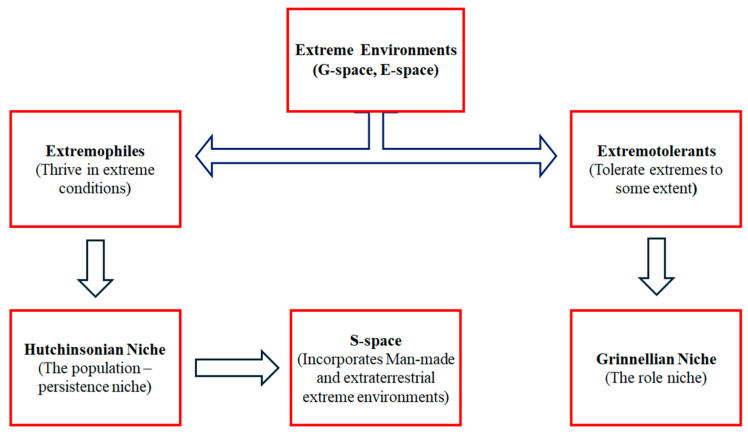
A simplified scheme that presents the differences between extremophiles and extremotolerant organisms while integrating niche theories and new concepts like S-space.

The question of whether we should approach extremophile microorganisms as de facto organisms or whether extreme conditions create or bring out extremophile organisms is crucial to theorising the concept. The distinction between extremophiles and extremotolerants could be used as a starting point towards this direction. As aforementioned, “extremophile” is used to describe organisms that have managed to not only survive but thrive at the most extreme ranges of environmental variables on Earth, while “extremotolerants” are organisms that tolerate them to a certain extent [121]. In terms of the Hutchinsonian niche, extremophiles in extreme conditions maintain stable populations in a region and can reproduce, rather than just existing as individual organisms. This means that true extremophiles have a broader Hutchinsonian niche, which is a characteristic of the species itself. On the other hand, extremotolerant organisms are associated with the Grinnellian niche, which is more about environmental factors in a geographic space where long-term population survival is not necessary. In this concept, there are extreme environments in the geographical space (G-space) found on Earth and extreme conditions in the environmental space (E-space) which do not match any locations on Earth. For each point in G-space, there is exactly one corresponding point in E-space, but each point in E-space may have multiple corresponding points in G-space or none at all. These extreme conditions can be either man-made simulations or real extraterrestrial environments, leading to the creation of another space, S-space. S-space expands the Hutchinsonian niche, incorporating both man-made and extraterrestrial conditions as part of a species’ possible environmental variables (Figure 1).

Recent experimental approaches have shown that common organisms, such as the eukaryotic green alga *Chlorella vulgaris*, can survive in extreme conditions unknown in terrestrial environments without the required adaptation. Exposure to an anoxic atmosphere consisting of 100% CO_2_ combined with extremely low atmospheric pressure (<10 mbar) (resembling a Mars-like atmosphere) differentiates the functional profile of the microalgae and allows them to survive in these conditions [122]. These findings reveal that the species’ potentialities are much broader than the Earth’s edges and can be expanded in space environments, and confirm the need for expanding the niche space by incorporating S-space into it.

In addition, this S-space is in agreement with the niche issues of lichens. Microalgae and fungi, which are also part of the lichen micro-ecosystem, are sensitive to environmental changes, but when they form lichens, they exhibit extremophilic behaviour, including resistance to absolute dryness for long periods of time [88], extremely low (−196 °C) and high (+70 °C) temperatures [89], and extreme UVB radiation [89], maintaining their metabolism and their ability to produce large amounts of H_2_ [88,89], which can be used as an energy source. Lichen symbiosis (or lichen micro-ecosystem) indicates mutualist-mediated niche expansions in extraterrestrial extreme environments [123] and also solidifies the need for the S-space.

Of course, the species’ potentialities in extreme environments should be taken into consideration within the BAM (Biotic, Abiotic, Mobility) concept [124], which constitutes both a conceptual framework and a methodological guide. The BAM concept explicitly incorporates Biotic, Abiotic, and Mobility factors as subsets of the niche [125]. As such, it attempts to disentangle the effects these factors have on the distributions of species. Each of the three components restricts the realised distribution of a species in different ways, and the relative structure of the three subsets needs to be taken into consideration when trying to predict the extremophilic or extremotolerant identity and/or the presence of species in extreme environments. Moreover, recent results have shown that the exposure of (micro)organisms to an absolute hydrogen atmosphere (100% H_2_) leads to reversible metabolic arrest, allowing the organisms to tolerate exposure to extreme conditions without functional effects for a long time, thus giving a new perspective to the panspermia theory [126].

All these reconsiderations open up new avenues for astrobiotechnological applications on other planets and expand the niche concept, incorporating the space prospect.

## 5. Conclusions

In a little more than 50 years, extremophiles have become an integral part of numerous research fields, including, but not limited to, microbiology, biochemistry, biotechnology, and astrobiology. Moreover, they are steadily claiming a central role in fields of the industrial world, namely the enzyme, biomining, and pharmaceutical industries. By conducting research on extremophiles, we not only deepen our understanding of every aspect of biotechnology and astrobiology that involves them, but we also reveal that species’ potentialities extend far beyond Earth’s boundaries. Thus, this study expands the n-hypervolume of the Hutchinsonian niche, incorporating both man-made and extraterrestrial conditions, the S-space. Consequently, research on extremophiles brings us significantly closer to unravelling lifelong questions that have been tantalising humanity such as “Where and how did life originate?” and “Are we alone in the universe?”

## Data Availability

Not applicable.
